# Learning to Lie: Effects of Practice on the Cognitive Cost of Lying

**DOI:** 10.3389/fpsyg.2012.00526

**Published:** 2012-11-30

**Authors:** B. Van Bockstaele, B. Verschuere, T. Moens, Kristina Suchotzki, Evelyne Debey, Adriaan Spruyt

**Affiliations:** ^1^Faculty of Psychology and Educational Sciences, Department of Experimental Clinical and Health Psychology, Ghent UniversityGhent, Belgium; ^2^Faculty of Social and Behavioural Sciences, Department of Clinical Psychology, University of AmsterdamAmsterdam, Netherlands; ^3^Faculty of Psychology and Neuroscience, Maastricht UniversityMaastricht, Netherlands

**Keywords:** deception, cognitive training, response inhibition, lie detection, intentionality

## Abstract

Cognitive theories on deception posit that lying requires more cognitive resources than telling the truth. In line with this idea, it has been demonstrated that deceptive responses are typically associated with increased response times and higher error rates compared to truthful responses. Although the cognitive cost of lying has been assumed to be resistant to practice, it has recently been shown that people who are trained to lie can reduce this cost. In the present study (*n* = 42), we further explored the effects of practice on one’s ability to lie by manipulating the proportions of lie and truth-trials in a Sheffield lie test across three phases: Baseline (50% lie, 50% truth), Training (frequent-lie group: 75% lie, 25% truth; control group: 50% lie, 50% truth; and frequent-truth group: 25% lie, 75% truth), and Test (50% lie, 50% truth). The results showed that lying became easier while participants were trained to lie more often and that lying became more difficult while participants were trained to tell the truth more often. Furthermore, these effects did carry over to the test phase, but only for the specific items that were used for the training manipulation. Hence, our study confirms that relatively little practice is enough to alter the cognitive cost of lying, although this effect does not persist over time for non-practiced items.

## Introduction

Cognitive theories on deception posit that deliberate and successful lying requires more cognitive resources than telling the truth (Vrij et al., 2006, 2011). Liars have to fabricate a story, monitor the reactions of the interaction partner, make sure that their story remains coherent and consistent, control behaviors that may signal lying or stress, and inhibit or conceal the truth. Several neuroimaging studies have provided evidence in line with this idea, showing that prefrontal brain regions which are involved in cognitive control (i.e., the anterior cingulate, dorsolateral prefrontal, and inferior frontal regions) are more active when participants are lying compared to when they are telling the truth (for reviews, see Christ et al., 2009; Gamer, 2011). The higher activation of brain regions involved in cognitive control suggests that individuals who are lying are engaged in a cognitively demanding task. Although it is generally agreed that lying comes at a cognitive cost and telling the truth is the default, dominant response, there is less agreement as to whether this cognitive cost is invariable or whether it is malleable through practice. For instance, pathological liars lie so often that lying becomes an automatism rather than an exception (Dike et al., 2005). One can thus expect that such people experience less cognitive difficulty when lying. The same holds for crime suspects who face interrogation and who have thoroughly practiced their story (Spence et al., 2008) or for people who have told the same lies so often that they believe their lies to be the truth (Polage, 2012).

To date, however, evidence concerning the effect of practice on the cognitive cost of lying is both scarce and mixed. Johnson et al. (2005) asked participants to memorize a list of words, and later used these and other words in an old/new recognition task. Over different blocks of the word recognition task, participants were instructed to either respond truthfully or deceptively. Crucially, they found that both behavioral and neurological measures of cognitive control were unaffected by practice in deceptive responding. These findings led the authors to conclude that lying *always* comes at a cognitive cost, and thus that the cognitive complexity of lying is resistant to practice. It should be noted, however, that Johnson et al. adopted a blocked within-subjects design with a random succession of truthful and deceptive blocks. Such an approach may have been suboptimal to study the impact of practice on the cognitive cost of lying as participants’ ability to lie in deceptive blocks may have been counteracted by intermediate truthful blocks, and vice versa. Vendemia et al. (2005) used autobiographical statements about their participants which were either true or false. In three sessions, participants were required to respond truthfully on half of the trials and deceptively on the other half of the trials, depending on the color of the statements. Although reaction time data revealed no practice effect whatsoever the errors data did show that the difference between deceptive and truthful responses diminished following practice. This latter finding illustrates that practice may have had some effect on the cognitive cost of lying. Furthermore, the training manipulation in this experiment was relatively weak, as participants were required to respond both truthfully and deceptively 50% of the time. As such, participants were not explicitly trained to either lie or tell the truth. Finally, using a Sheffield lie test (Spence et al., 2001; a variant of the differentiation of deception paradigm by Furedy et al., 1988), Verschuere et al. (2011b) recently challenged the idea that the cognitive cost of lying is resistant to practice. In the standard version of this task, autobiographical questions are presented on a computer screen, and participants provide yes/no-answers using one of two different response keys. The questions can appear in two different colors, and participants are instructed to lie if the sentence is presented in the one color (lie-trials) and to tell the truth if the sentence is presented in the other color (truth-trials). In a control group, with 50% lie-trials and 50% truth-trials, they found that lying requires more cognitive resources than telling the truth, as illustrated by slower response times and more errors on lie-trials compared to truth-trials (i.e., the lie-effect; see also Sartori et al., 2008). In two other groups, a number of filler trials were added in order to manipulate the numbers of lie and truth-trials. In the frequent-lie group, all these filler trials required a deceptive response. In contrast, all the filler trials required a truthful response in the frequent-truth group. As such, participants in the frequent-lie group were required to lie on 75% of the trials whereas participants in the frequent-truth group only lied on 25% of the trials. Both the response latency data and error rates indicated that lying became easier while people were lying more often, and lying became more difficult while people gave more truthful responses.

In the present experiment, we further examined the influence of practice on the cognitive cost of lying. More specifically, we investigated (1) whether practice has an effect on participants’ initial cognitive cost of lying, and (2) whether such effects continue to exist after the training, and thus whether practice really changes the dominance of the truth response. The experimental design used by Verschuere et al. (2011b) did not allow firm conclusions concerning this important matter because they only assessed the lie-effects while participants were being exposed to either a high proportion of lie-trials or a high proportion of truth-trials. As such, their results indicate only that *while* participants are lying often, lying becomes easier, and *while* participants are often telling the truth, lying becomes more difficult. Therefore, we attempted to replicate and extend the findings of Verschuere et al. by prolonging the training phase and by adding a baseline phase and a test phase in which participants were required to respond deceptively and truthfully equally often. We expected that (1) the cognitive cost of lying would change as a result of our training manipulation, and (2) if practice does genuinely change the dominance of the truth response, the change in the cognitive cost of lying would persist over time. In other words, we expected a linear trend in the size of the lie-effect in the training phase as a function of the proportion of lie-trials, with a smaller lie-effect in the frequent-lie group, a medium lie-effect in the control group, and a larger lie-effect in the frequent-truth group. If training genuinely alters the dominance of the truth response, this linear trend should extend to the test phase.

## Materials and Methods

### Participants

Forty-five undergraduate students (23 men) of Ghent University participated in exchange for course credits. The data of three participants were not analyzed because of poor accuracy on test trials (see below; participants’ accuracy scores = 54, 58, and 62%; deviating more than 2.5SDs from the group average = 89%, SD = 10%). Hence, our results are based on the data of 42 participants. All participants provided written informed consent prior to the experiment.

### Modified Sheffield lie test

In the Sheffield lie test, we used 108 different questions. Thirty-six of these questions were yes-or-no questions about basic semantic knowledge, and were used to allow us to give performance feedback during the acquaintance phase. Half of these practice questions required a “no”-response (e.g., “Is London a Belgian city?”), and the other half required a “yes” -response (e.g., “Is a stone hard?”). The remaining 72 questions were autobiographical yes-or-no questions related to actions that participants had or had not performed on the day of testing (see Table A1 in Appendix). Before the start of the experiment, participants were asked to give a truthful response to these questions. Some of these questions were more likely to elicit an affirmative response (e.g., “Did you drink water?”) than others (e.g., “Did you greet a police officer?”). In this way we tried to establish a yes-no ratio of approximately 50%. Analyses of the yes/no ratio revealed that participants gave more no-answers (67%, SD* *= 6.92) than yes-answers (33%), *t*(41) = 15.82, *p* < 0.001. However, this was the case for all three groups (see below), all *t*s > 9.27, all *p*s < 0.001, and there was no difference between the groups, *F*(2, 39) = 1.65, *p* = 0.20. Half of these questions were used in filler trials, and the other half in test trials (counterbalanced). The general appearance of test trials and filler trials was identical. On each trial, a sentence was presented in white bold Arial font in the center of the black screen, together with the response labels “YES” and “NO” at the sides of the screen. The response labels could either appear in blue or in yellow, and, depending on this color, participants were required to give a truthful or a deceptive yes-no response (i.e., blue = truth, yellow = lie, or vice versa) by pressing either the “4” or the “6” key on the numeric pad of a standard AZERTY keyboard. The assignment of the two response buttons to either yes-or-no responses was counterbalanced across participants, as was the assignment of the two colors to either truthful or deceptive responding. There was no response deadline. In order to prevent strategic recoding of the task, we also included catch trials (Johnson et al., 2003, 2005; Verschuere et al., 2011b). On these trials, either the word “yes” or the word “no” appeared in the center of the screen and participants were required to respond according to their meaning (i.e., press the yes-button for the word “yes”, and the no-button for the word “no”), irrespective of the color of the response labels.

Our modified version of the Sheffield lie test consisted of 924 trials, presented in an acquaintance phase (24 trials), a baseline phase (180 trials), a training phase (540 trials), and a test phase (180 trials). The acquaintance phase consisted of only semantic trials with performance feedback allowing participants to familiarize with the task at hand. The data of these trials were not analyzed. In the *baseline* phase, we presented 72 test trials (36 truth, 36 lie), 72 filler trials (36 truth, 36 lie), and 36 catch trials (18 yes, 18 no) in an intermixed, random fashion. In the *training* phase, participants were randomly assigned to one of three different groups. In all three groups, we presented three identical blocks, each consisting of 72 test trials (36 truth, 36 lie), 72 filler trials, and 36 catch trials. For the test trials, the proportion of truthful and deceptive responses remained 50/50. However, the proportion of filler trials requiring a deceptive or a truthful response differed across the three groups in the training phase. In the frequent-lie group, all the filler trials required a deceptive response, whereas in the frequent-truth group, all the filler trials required the participants to respond truthfully. Finally, in the control group, half of the filler trials required a truthful response, and half required a deceptive response. As such, due to the manipulation of the lie-truth proportion of the filler trials, participants in the frequent-lie group lied on 75% of the trials in the training phase, whereas participants in the frequent-truth group only lied on 25% of the trials. Participants were not informed about this manipulation. The last phase was a *test* phase, which was identical to the baseline phase. Participants were allowed to take a short break after each block.

### Data processing

For the analyses of the response latency data, trials with erroneous responses were discarded. In order to reduce the impact of extreme reaction times, we recoded response latencies faster than 300 ms and slower than 3000–300 and 3000 ms respectively (Greenwald et al., 1998)^1^. Using this procedure, we recoded a total of 10.43% of the correct trials (for latencies larger than 3000 ms: 5.43% lie-trials and 4.77% truth-trials; for latencies smaller than 300 ms: 0.11% lie-trials and 0.12% truth-trials). Next, for each participant and each experimental condition, we calculated the average response latencies (ms) and accuracy scores (%). Finally, we calculated “lie-effect” scores by subtracting the response latencies and accuracy scores on lie-trials from the response latencies and accuracy scores on truth-trials. A large positive lie-effect score reflects greater difficulty in lying, and a negative lie-effect score reflects greater ease in lying. For all analyses, the alpha level was set to 0.05.

## Results

### Test trials

Test trials were analyzed in order to investigate (1) whether practice in truthful and deceptive responding on the filler trials affected truthful and deceptive responding on the test trials during the training phase, and (2) whether these effects transferred to the test phase. To do so, we subjected the lie-effect scores of both the reaction time data and the errors to 3 (Group: frequent-lie vs. control vs. frequent-truth) × 3 (Experiment Phase: baseline vs. practice vs. test) repeated measures ANOVAs with Group as a between subjects factor and Experiment Phase as a within subject factor. We expected no group differences in the baseline phase, and a linear effect of Group (i.e., a gradual increase in the magnitude of the lie-effect from the frequent-lie group over the control group to the frequent-truth group) in the training phase. Finally, we expected this training effect to generalize to the test phase.

#### Reaction times

**Figure 1:**
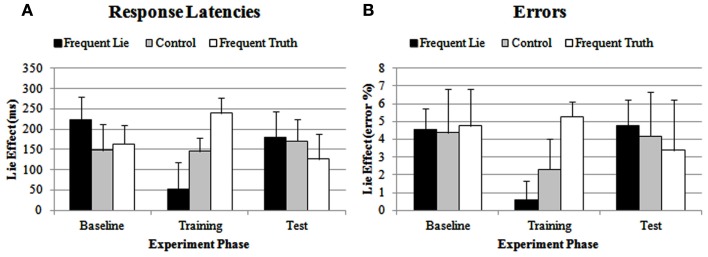
**Lie-effect of response latencies (A) and errors (B) on test trials for the frequent-lie, control, and frequent-truth group, during the baseline phase, the training phase and the test phase**. Error bars represent the standard error of the mean.

Neither of the main effects reached significance, both *F*s < 1. However, the interaction between the linear effect of Group and Experiment Phase followed a significant quadratic course, *F*(1, 39) = 18.56, *p* < 0.0005 (see Figure 1A), indicating that the linear effect of Group varied across the three Experiment Phases. Whereas the lie-effect was the same for the three groups during the baseline phase, *F* < 1 (frequent-lie group: *M* = 222, SD = 217; control group: *M* = 148, SD = 242; frequent-truth group: *M* = 163, SD = 176), there was a significant linear effect of Group during the training phase, *F*(1, 39) = 7.56, *p *< 0.01, *f* = 0.44^2^. This linear course illustrates that the size of the lie-effect gradually increased from the frequent-lie group (*M* = 53, SD = 245) over the control group (*M* = 145, SD = 123) to the frequent-truth group (*M* = 239, SD = 146). In the test phase, there was no effect of Group, *F* < 1, indicating that the lie-effect scores of the three groups no longer differed significantly (frequent-lie group: *M* = 180, SD = 238; control group: *M* = 171, SD = 195; frequent-truth group: *M* = 127, SD = 231).

#### Errors

As for the reaction time data, neither of the main effects reached significance, both *F*s < 1.45, both *p*s > 0.24. However, the interaction between Experiment Phase and the linear effect of Group again followed a significant quadratic course, *F*(1, 39) = 4.73, *p* < 0.05 (see Figure 1B), illustrating that group differences in the lie-effect varied across the three Experiment Phases. The lie-effect was the same for the three groups in the baseline phase, *F* < 1 (frequent-lie group: *M* = 4.56, SD = 4.44; control group: *M* = 4.37, SD = 7.68; frequent-truth group: *M* = 4.76, SD = 9.27). However, in the training phase, there was a significant linear effect of Group, *F*(1, 39) = 7.21, *p* < 0.05, *f* = 0.42. As can be seen in Figure 1B, the lie-effect in the training phase was smaller in the frequent-lie group (*M* = 0.60, SD = 3.90), intermediate in the control group (*M* = 2.32, SD = 3.01), and larger in the frequent-truth group (*M* = 5.27, SD = 6.32). These group differences were no longer significant in the test phase, *F* < 1 (frequent-lie group: *M* = 4.76, SD = 5.49; control group: *M* = 4.17, SD = 10.77; frequent-truth group: *M* = 3.39, SD = 9.26).

### Filler trials

Filler trials were analyzed in order to investigate whether practice with specific items influences the cognitive cost of lying on these specific items. For these analyses, we discarded the data of the training phase because these trials were all either lie-trials or truth-trials for the two experimental groups, and hence do not allow to calculate the crucial difference between truthful and deceptive responses. For both reaction times and error rates, the lie-effect scores were subjected to a 3(Group) × 2(Experiment Phase: baseline vs. test) repeated measures ANOVA. We expected no group differences in the baseline phase, and a linear effect of Group (i.e., a gradual increase in the magnitude of the lie-effect from the frequent-lie group over the control group to the frequent-truth group) in the test phase.

#### Reaction times

**Figure 2:**
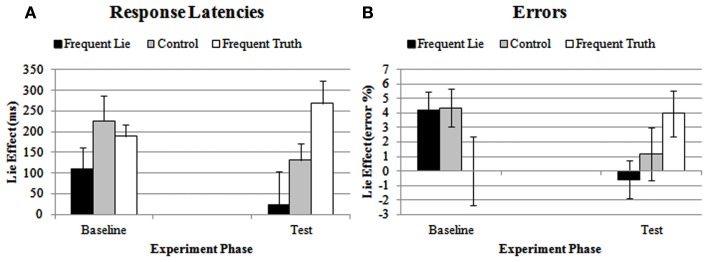
**Lie-effect of response latencies (A) and errors (B) on filler trials for the frequent-lie, control, and frequent-truth group, during the baseline phase and the test phase**. Error bars represent the standard error of the mean.

Analysis of the lie-effect scores on filler trials yielded a significant main effect of Group, *F*(2, 39) = 4.07, *p* < 0.05. Follow-up between-group comparisons showed that neither the frequent-truth group nor the frequent-lie group differed significantly from the control group, *F*(1, 26) = 0.95, *p* = 0.10, and *F*(1, 26) = 1.04, *p* = 0.32, respectively. However, the lie-effect was significantly larger in the frequent-truth group compared to the frequent-lie group, *F*(1,26) = 7.64, *p* < 0.05. The interaction between Experiment Phase and the linear effect of Group was not significant, *F*(1, 39) = 2.65, *p* = 0.11. Exploratory analyses on each experiment phase separately showed, however, that there was no linear effect of Group in the baseline phase, *F*(1, 39) = 1.29, *p* = 0.26 (frequent-lie group: *M* = 111, SD = 188; control group: *M* = 226, SD = 230; frequent-truth group: *M* = 189, SD = 102), but a clear linear effect in the test phase, *F*(1, 39) = 8.28, *p* < 0.005, *f* = 0.46. Figure 2A illustrates that in the test phase, the lie-effect in the test phase gradually increased from the frequent-lie group (*M* = 24, SD = 296) over the control group (*M* = 131, SD = 156) to the frequent-truth group (*M* = 269, SD = 199).

#### Errors

Neither of the main effects reached significance, both *F*s < 1, but the interaction between Experiment Phase and the linear effect of Group was significant, *F*(1, 39) = 6.27, *p* < 0.05 (see Figure 2B). A one-way ANOVA on the lie-effect scores revealed no linear effect of Group in the baseline phase, *F*(1, 39) = 2.88, *p* = 0.10 (frequent-lie group: *M* = 4.17, SD = 4.84; control group: *M* = 4.36, SD = 4.84; frequent-truth group: *M* = 0.00, SD = 8.92). In contrast, the main effect of Group showed a significant linear course in the test phase, *F*(1, 39) = 4.11, *p* < 0.05, *f* = 0.33. As can be seen in Figure 2B, the lie-effect again gradually increased from the frequent-lie group (*M* = −0.59, SD = 4.89) to the control group (*M* = 1.19, SD = 6.86), and from the control group to the frequent-truth group (*M* = 3.97, SD = 5.94).

## Discussion

In the present experiment, we investigated whether practice in lying or telling the truth influences the cognitive cost of lying. Like Verschuere et al. (2011b), we found that during the training phase, lying became more difficult for participants in the frequent-truth group than for participants in the frequent-lie group. As such, our present results are in conflict with the conclusions of Johnson et al. (2005) and Vendemia et al. (2005), who both argued that the cognitive cost of lying is resistant against practice. However, the experimental designs of both Johnson et al. and Vendemia et al. may have been suboptimal to investigate the effects of practice on the cognitive cost of lying. As mentioned earlier, Johnson et al. randomly intermixed blocks of truth-trials and blocks of lie-trials *within* participants, which may have prevented consistent changes in their participants’ abilities to lie. Likewise, participants in the experiment of Vendemia et al. were manipulated to lie on only 50% of the trials, mimicking the design that we used for our control group. It could therefore be argued that participants in the study of Vendemia et al. were not consistently trained to respond either truthfully or deceptively, making consistent changes in their cognitive ability to lie less likely.

Furthermore, we found that practice can have some enduring effects on the cognitive cost of lying in the test phase (see also Hu et al., 2012). During the test phase, lying was easier for participants in the frequent-lie group and lying was more difficult for participants in the frequent-truth group. However, these effects were limited to the data of the filler questions (i.e., the specific questions which were used for the training manipulation). The finding that the training effect did not carry over to the test questions (i.e., the questions which required 50% truthful and 50% deceptive responses throughout the entire experiment) indicates that our training manipulation was not sufficient to genuinely alter the dominance of the truth response. Although unexpected, it is not uncommon to find that cognitive training manipulations do not generalize to non-trained stimuli (e.g., Schoenmakers et al., 2007). Further research is needed to investigate whether a more intensive training (e.g., over several days) would have such an enduring effect on new items. Nevertheless, our finding that changes in the lie-effect on the filler trials persisted over time further challenges the assumption that “… even after thousands of trials of practice, it is unlikely that the increased difficulty associated with making deceptive responses will be erased entirely” (Johnson et al., 2005, p. 402).

An interesting remaining issue concerns the mechanism underlying the changes in the lie-effect on test trials during the training phase. As this change did not persist in the test phase, the effects may be caused by specific properties of the task or the design. In our opinion, there are at least three – not necessarily mutually exclusive – possible explanations. A first explanation stems from research on task switching (e.g., see Monsell, 2003; Kiesel et al., 2010; Vandierendonck et al., 2010). In a typical task switching design, participants are on each trial required to perform one out of two different tasks. Participants are generally faster and more accurate on trials that are preceded by a trial in which the same task was performed compared to trials that are preceded by a trial in which the other task was performed. The drop in performance on trials that require a task switch is known as the switch cost. Our present experiment bears some resemblance with such dual task paradigms in the sense that our participants were also required to perform one out of two possible tasks, namely lying or telling the truth. Crucially, during the training phase, the switching between lying and telling the truth differed between the three groups. In the frequent-truth group, the overall larger proportion of truth-trials increased the probability that truth-trials involved repetitions and that lie-trials involved a switch (e.g., Truth-Truth-Truth-Lie-Truth). In a similar fashion, in the frequent-lie group, truth-trials were more likely to involve switches and lie-trials were more likely to involve repetitions (e.g., Lie-Lie-Truth-Lie-Lie). Thus, task switch costs may have increased the lie-effect in the frequent-truth group, and reduced it in the frequent-lie group. A second possible mechanism behind the group differences on the test trials during the training phase is the oddball-effect (e.g., see Squires et al., 1975; Stevens et al., 2000; Goldstein et al., 2002). In a typical oddball task, participants are required to respond differently to two types of stimuli. Crucially, one of the stimuli is highly frequent, and the other is less frequent. Participants are typically fast to respond to the frequent stimuli, but slower to respond to the less frequent stimuli. In the present study, participants in the frequent-lie group encountered many lie-trials, and truth-trials were relatively rare. As a result, these participants were more likely to respond fast on lie-trials and slow on truth-trials, resulting in a decreased lie-effect. Inversely, in the frequent-truth group, the truth-trials were highly frequent and the lie-trials were relatively rare, resulting in fast responses on truth-trials and slower responses on lie-trials, and thus leading to a stronger lie-effect. A third possible explanation for the group differences during the training phase is goal neglect (e.g., see De Jong et al., 1999; Kane and Engle, 2003; Debey et al., 2012). According to the goal neglect theory (Duncan, 1995), the selection of an appropriate response is guided by task goals. The more active such a task goal is, the more accurate and fast a response will be, while responses will be slower and less accurate if they are guided by a more neglected task goal. It is possible that our manipulation of the proportions of lie and truth-trials resulted in our three groups having different dominant task goals. As a result, in the frequent-lie group, the most active task goal may have been to respond deceptively and inhibit truthful responses, resulting in fast and accurate responses on lie-trials and slower and less accurate responses on truth-trials, resulting in a smaller lie-effect. Inversely, if the main task goal in the frequent-truth group was to respond truthfully and avoid lying, this would result in fast and accurate responses on truth-trials, and slower and less accurate responses on lie-trials, hence resulting in a larger lie-effect.

In future research, it may be possible to differentiate between these different mechanisms. For instance, presenting lie- and truth-trials in a predictable order should reduce the impact of a switch cost or oddball-effect, while such a manipulation is unlikely to influence the effect of goal neglect. Another possibility is to manipulate the duration of the response-stimulus interval (RSI). While longer RSIs provide more preparation time and should hence decrease switch costs (Monsell, 2003), longer RSIs have also been shown to hamper goal maintenance and induce goal neglect (De Jong et al., 1999; Debey et al., 2012). Thus, if the difference in the lie-effects during the training phase is driven by a switch cost, then an increased RSI should reduce this difference. Alternatively, if the difference is driven by goal neglect, then an increased RSI should further inflate the effect.

The results of our study may also have implications for the detection of deception in forensic settings (Granhag and Strömwall, 2004; Verschuere et al., 2011a). Given the fact that lying becomes more difficult while people often tell the truth, the accuracy of lie detection tests may be improved by adding a large number of verifiable questions to the interrogation. Our results suggest that if suspects are obliged to respond truthfully on these verifiable questions, they may experience greater difficulty when lying on a crucial incriminating question. Our results of the filler trials suggest that the cognitive cost of lying can be reduced for specific well-trained lies. Translated to the forensic context, this may mean that a guilty suspect who has repeated the same lies over and over again (e.g., to the police, to lawyers, to judge, etc.) may experience less cognitive load when lying. Our results suggest that, with repeated lying, deceptive responses may cognitively mirror truth telling, thus hampering lie detection. However, more research is needed to investigate these forensic implications in detail. For instance, it is uncertain whether our results would be replicated in a context where participants have something to gain or lose or where arousal and emotional distress are high, or whether certain strategies or counter-measures can influence our pattern of results.

Our study also has a number of limitations. First, our sample consisted only of 42 participants, resulting in 14 participants per group. As a result, our experiment may have lacked the statistical power that is needed to uncover smaller effects. A second limitation is more inherent to our specific methodology, namely our use of autobiographical questions as stimuli in the Sheffield lie test. As mentioned earlier, these specific questions were not emotionally salient, nor were they related to crime. As such, the possible forensic implications of our present results need to be addressed in an ecologically more valid context, for instance by using questions related to a mock crime that participants have or have not committed. Also, although the autobiographical questions that we used were related to actions that participants had or had not performed on the day of testing, some participants may have been uncertain of their initial answers as well as their responses during the subsequent Sheffield lie test. Such uncertainty may have arisen especially on questions concerning habitual behaviors (e.g., buying a newspaper), or on actions that were performed on the day before testing. In our follow-up research, we now ask participants to perform specific actions in the laboratory, prior to testing (e.g., see Debey et al., 2012). This new methodology has advantages over the methodology that we used in the present experiment, as it allows us to control the yes/no ratio of answers and it reduces possible uncertainty with the participants.

In sum, the data of our present experiment suggest that lying becomes cognitively less demanding while participants are often lying, and that lying becomes cognitively more difficult while participants are often telling the truth. Furthermore, these effects were not due to baseline differences between the three groups, and practice on specific lies had enduring effects over time, suggesting that the detection of well-trained lies may prove to be a thorny issue.

## Conflict of Interest Statement

The authors declare that the research was conducted in the absence of any commercial or financial relationships that could be construed as a potential conflict of interest.

## Footnotes

^1^Another outlier analysis in which we first removed all reaction times faster than 200 ms and slower than 5000 ms and then removed all reaction times that deviated more than 3SDs from the individual’s mean yielded the same overall pattern of results.

^2^We calculated the effect size *f* using the following formula: $f=\surd \left[\eta_{P}^{2}/\left(1-\eta_{P}^{2}\right)\right]$. According to Cohen (1992), values from 0.10 represent small effects, values from 0.25 represent medium effects and values from 0.40 represent large effects.

## Appendix

**Table A1:** **List of the two lists of autobiographical questions that were used as either filler or test items (counterbalanced) in the experiment**.

List 1	List 2
Did you go for a run?	Did you stop at a traffic light?
Did you go down a staircase?	Did you go to a supermarket?
Did you go up a staircase?	Did you buy some flowers?
Did you buy petrol?	Did you do the dishes?
Did you eat chocolate?	Did you take an elevator?
Did you take a bus?	Did you clean a window?
Did you take a train?	Did you reschedule an appointment?
Did you open a dustbin?	Did you read a book?
Did you take a bath?	Did you park a moped?
Did you make a sandwich?	Did you squeeze a lemon?
Did you post a letter?	Did you send an e-mail?
Did you close a door?	Did you stroke a pet?
Did you take a shower?	Did you wear a coat?
Did you buy a newspaper?	Did you open a fridge?
Did you buy a magazine?	Did you switch on a computer?
Did you use a knife?	Did you smoke a cigarette?
Did you use an umbrella?	Did you look at a watch?
Did you take a pill?	Did you open a water tap?
Did you speak to a police officer?	Did you lift a toilet seat?
Did you eat a grapefruit?	Did you use a pedestrian crossing?
Did you break a window?	Did you use an ATM?
Did you use a telephone?	Did you change money?
Did you receive a telegram?	Did you vacuum a carpet?
Did you drink fruit juice?	Did you drink cough syrup?
Did you listen to the radio?	Did you greet someone?
Did you use the internet?	Did you clean the house?
Did you stand in a queue?	Did you check your PO box?
Did you sit in a waiting room?	Did you brush your teeth?
Did you make your bed?	Did you listen to an MP3?
Did you wash your hands?	Did you sit on a bicycle?
Did you sign a document?	Did you stand on a ladder?
Did you drink coffee?	Did you sit on a chair?
Did you speak to a child?	Did you rip a piece of paper?
Did you watch television?	Did you water the plants?
Did you eat onions?	Did you use your keys?
Did you drink water?	Did you boil some water?
